# Improvement in Overall Survival from Extremity Soft Tissue Sarcoma over Twenty Years

**DOI:** 10.1155/2015/279601

**Published:** 2015-03-03

**Authors:** Andrew J. Jacobs, Ryan Michels, Joanna Stein, Adam S. Levin

**Affiliations:** ^1^Hofstra North Shore-LIJ School of Medicine, 500 Hofstra University, Hempstead, NY 11549, USA; ^2^Department of Orthopaedics, North Shore Long Island Jewish Medical Center, 270-05 76th Avenue, New Hyde Park, NY 11040, USA; ^3^Biostatistics Unit, Feinstein Institute for Medical Research, 350 Community Drive, Manhasset, NY 11030, USA; ^4^Department of Orthopaedic Surgery, Johns Hopkins University, 601 N. Caroline Street, Baltimore, MD 21287, USA

## Abstract

Several patient demographic factors, including marital status, have been demonstrated to have prognostic significance for survival in extremity soft tissue sarcoma (ESTS). A study population of 12,546 adult patients diagnosed with ESTS from 1991 to 2010 was identified from the SEER database, a large population-based registry, in order to determine whether overall survival had changed over this recent 20-year period. The study population was divided into three groups by year of diagnosis: 1991–1996, 1997–2003, and 2004–2010. We used the Kaplan-Meier method and Cox proportional hazards regression to assess survival differences between different demographic groups and prognostic clinical characteristics. Over the course of time, the 5-year overall survival rates have increased from 28% in the earliest time period to 62% in the latest (*P* < 0.0001). On multivariate analysis, the mortality rate progressively declined from the 1991–1996 group (HR: 3.02, CI: 2.78–3.29) to the 1997–2003 group (HR: 2.21, CI: 2.06–2.37), with the 2004–2010 group having the best overall survival, despite increases in the proportion of patients with tumors greater than 5 cm in size (*P* < 0.0001), and those presenting with metastasis (*P* < 0.0001).

## 1. Introduction

Extremity soft tissue sarcomas (ESTS) comprise a collection of rare mesenchymal malignancies [1]. These represent a histologically heterogeneous group of diseases arising from connective tissues, many of which present a high risk of distant metastasis [2–4]. Surgical resection is the mainstay of current treatment approaches, with limb salvage considered a clinically appropriate objective for most tumors [5]. Adjuvant or neoadjuvant radiation has been recognized to reduce the rate of local recurrence, particularly in high-grade tumors. However, there remains a question as to whether radiation therapy may increase overall survival in patients with high-grade soft tissue sarcomas [6–10].

Due to the low incidence of ESTS, most prior studies of this disease have been small and from a single institution [11–15]. Contemporary population-based studies of ESTS have demonstrated clinical and demographic patient factors that may be associated with prognosis [16, 17].

The National Cancer Institute's Surveillance, Epidemiology, and End Results (SEER) database provides a mechanism by which to analyze this disease through a cross section of the United States' population [18]. The goal of this study was to assess incidence and survival of ESTS during a 20-year period, from 1991 to 2010, to determine if there have been changes in the overall survival from this disease.

## 2. Methods

The SEER program is developed by the National Cancer Institute (NCI) for evaluation of population-based cancer statistics in the United States. The database comprises 18 geographic registries, covering approximately 28% of the United States' population [19]. Together, these registries encompass the demographic diversity of the US population, with comparable socioeconomic and racial representation relative to the national population [20]. Every case of cancer within the registries' geographic domains is recorded [21].

This database was used to identify a cohort of 15,382 adult patients, age 17 and older, diagnosed with ESTS from 1991 to 2010. Inclusion criteria were diagnosis of sarcoma, primary to connective, subcutaneous, and other soft tissues of the lower limb and hip, or upper limb and shoulder. Patients diagnosed in 2004 or later who had an unknown AJCC Stage, histologic grade, CS tumor size, lymph node involvement, extension, and metastasis at the time of diagnosis were excluded, as were patients diagnosed prior to 2004 whose data were incomplete regarding histologic grade, EOD tumor size, lymph node, and extent. These exclusions yielded a study population of 12,546 patients.

The study population was evaluated based upon demographic characteristics, including age, race, Hispanic ethnicity, sex, marital status, year of diagnosis, and geographic location. Prognostic tumor characteristics that were examined include tumor size, anatomic site (upper versus lower extremity), metastatic disease at presentation, tumor grade, and type of sarcoma. AJCC Stage was only included in the SEER database after 2004. The use of radiation therapy and surgery were included in the analysis as well.

Patients were grouped by year of diagnosis for comparison: 1991–1996, 1997–2003, and 2004–2010. For demographic analysis, race was categorized as White, Black, or Asian/other. Marital status was categorized as single (never married), married (including common law), other (including separated, divorced or widowed), or unknown. The rural-urban continuum code was collapsed into a binary variable: Metro county or Non-Metro county, using guidelines by SEER and ERS [22, 23]. The SEER registries were aggregated into geographic regions: Northeast, South, Southwest, Midwest, and West.

Extracted data was used to categorize tumor size into ≤5 cm, >5 cm, or unknown, and categorize lymph node involvement, extension of the tumor, and presence of metastasis at the time of diagnosis. ICD-O-3 histologic types were collapsed into the following categories: fibromatous connective tissue neoplasm, myxomatous connective tissue neoplasm, lipomatous, myomatous, synovial connective tissue neoplasm, ESTS not otherwise specified (NOS), and all other types, including osteosarcoma, chondrosarcoma, vascular tumors, and Ewing sarcoma.

Incidence rates were analyzed using SEER∗Stat (version 8.1.5; NCI, Bethesda, MD). Incidence rates were age-adjusted to the 2000 US Standard Population. Annual percent change was calculated using the weighted least squares method, with the Tiwari modification used for confidence intervals.

Statistical analysis was performed using SAS version 9.3 (SAS Institute, Cary, NC). Survival time was measured in years. Chi-square tests were performed to assess differences between time periods on demographic and clinical factors. Comparisons of survival time among categorical grouping variables were accomplished by the computation of Kaplan-Meier product-limit curves, with the effects of categorical demographic, clinical, pathologic, and treatment variables assessed using the log-rank test. The Bonferroni test was used when performing multiple comparisons. Cox proportional hazards regression was used to estimate survival differences for continuous variables.

Factors that appeared to be significantly associated with survival in univariate analysis were considered for inclusion in the Cox proportional hazards regression multivariable model. A result was considered statistically significant with a *P* value <0.05. Efron's method was used to adjust for tied failure times.

## 3. Results

A majority of patients in this study were 60 years of age or older (50%), Caucasian (82%), and married (60%). The population was 54% male and 46% female. The most common histologic diagnoses were fibromatous (33%) and lipomatous (24%). The least common was myxomatous (0.81%). Tumors occurred more frequently in the lower limb (74%) than the upper limb (26%).

**Figure 1:**
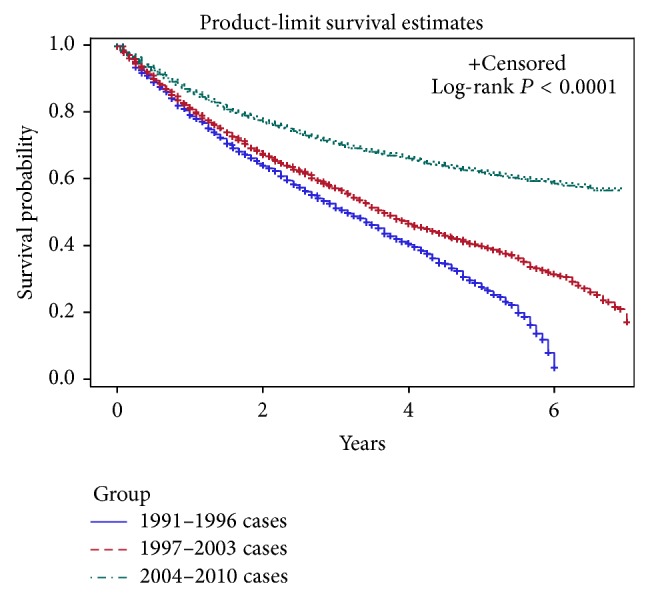
Kaplan-Meier product-limit curve of overall survival of ESTS from 1991–2010.

**Figure 2:**
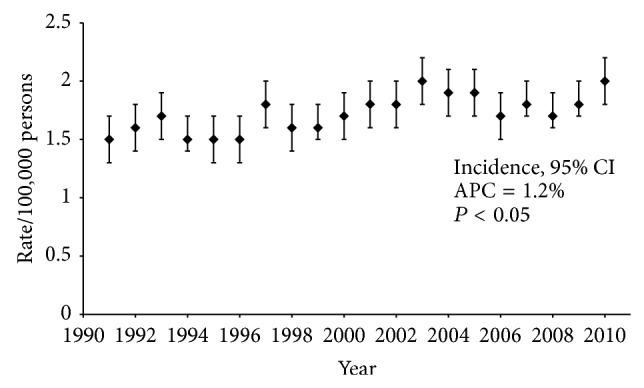
Incidence of ESTS in the United States, age-adjusted to the 2000 US Standard Population.

The 5-year overall survival of ESTS improved progressively during the study period, from 28% in 1991–1996, to 40% in 1997–2003, to 62% in 2004–2010 (Figure 1). There was a significant difference between these survival curves (*P* < 0.0001). The incidence of ESTS increased slightly during the study period, from 1.5/100,000 in 1991, to 2.0/100,000 in 2010. This represents an annual percent change of 1.2% (Figure 2).

The clinical picture of the adult ESTS population also changed significantly during the 20-year study period (Table 1). The population became older, with the proportion of patients 60-years of age and older increasing modestly between time periods, from 49.7% to 52.0% (*P* < 0.0001). Similarly, the proportion of patients under the age of 30 decreased from 9.2% to 6.8% (*P* < 0.0001).

Among patients with known disease grade, the patient population in 2004–2010 presented with more advanced disease than did patients in 1991–1996, with the proportion of patients presenting with high-grade tumors increasing from 51.8% to 61.2%. Among patients whose metastatic status at presentation was known, there was an increase in the percentage of patients who presented with metastasis over the course of the study, from 9.5% in the earliest time period to 14.9% in the most recent. Similarly, the proportion of patients with tumors >5 cm increased from 60.4% to 66.7% over the study period. These aforementioned differences between time periods demonstrated statistical significance (Table 1). Anatomical tumor location changed significantly throughout this study period as well, with the proportion of lower limb tumors increasing from 71.7% to 75.4% (*P* = 0.0034).

The effects of race, ethnicity, and county metropolitan status were not consistent throughout this study period. When comparing the univariate results (Table 2), Hispanic ethnicity was a significant factor only during the 1997–2003 time period, during which time Hispanic patients had improved overall survival over non-Hispanic patients (*P* = 0.0004). Race was not a significant factor during the 1991–1996 time period. However, African Americans had a decreased overall survival when compared to Caucasians in 2004–2010 (*P* = 0.0332). County metropolitan status was significant only during the 1997–2003 time period, during which patients in metro counties had increased overall survival compared to patients in non-Metro counties (*P* < 0.001).

While the percentage of patients treated with surgery remained constant, the use of radiation therapy increased during this study period, with the proportion of patients receiving radiation therapy increasing from 48.8% to 53.0% (*P* < 0.0001). This slight change in the use of neoadjuvant or adjuvant radiation temporally relates to the publication of two major randomized control trials during the initial study period.

On multivariate analysis, the mortality rate progressively declined from the 1991–1996 group (HR: 3.02, CI: 2.78–3.29), to the 1997–2003 group (HR: 2.21, CI: 2.06–2.37), with the 2004–2010 reference group, having the best overall survival, while adjusting for age, Hispanic ethnic status, registry region, marital status, county foreign born percent, percent with high school graduation, treatment with radiation and surgery, histologic type, metastatic disease status, tumor size, grade, and anatomic site (Table 3). Results from the multivariate analysis showed each of these factors to be independent predictors of survival, excluding anatomic site and registry region; though site and region were not statistically significant, these factors were kept in the final model due to clinical importance and improvement of model fit. Lastly, interaction between tumor size and anatomic site was also assessed, but the results were not statistically significant and are therefore not reported.

## 4. Discussion

Overall survival of ESTS improved over time in our 20-year study. This improvement in overall survival occurred despite increases in the proportion of older patients, as well as increases in the proportions of patients with large tumors and those with metastatic disease. ESTS is understood to have a bimodal age distribution [24]. It is therefore not surprising that as the US population ages, the proportion of older patients diagnosed with ESTS will increase. In fact, the current findings suggest that the overall incidence of ESTS may be increasing slightly over time. It is, however, surprising that despite increases in the proportion of patients with tumors >5 cm size, as well as those with metastatic disease at the time of diagnosis, overall survival improved with time. This is in contrast to the dogma that earlier detection of smaller tumors leads to increased survival in other cancers [25–28]. While this study cannot determine whether earlier detection may have influenced prognosis, these results indicate that the more recent study populations presented at an apparently later stage in the disease course. These findings may potentially be attributable to improvements in the ability for advanced imaging to detect distant metastases, though such an analysis is beyond the scope and limitations of the current analysis.

The use of radiation increased over the course of this study period, from 49% in 1991–1996 to 53% in 2004–2010. Most prior studies have failed to demonstrate an improvement in overall survival with radiation therapy [8, 29, 30]. Our results, however, demonstrate radiation therapy to be an independent prognostic indicator of overall survival. A previous analysis of the SEER database also demonstrated an association between radiation therapy and improved overall survival in patients with high-grade ESTS [6]. Given that the previous study likely included an overlapping study population to that in the current analysis, our findings may represent a confirmation of the prior analysis. Interestingly, in the current study, when the use of radiation was included in multivariate analysis, the year of diagnosis remained a significant predictor of overall survival. This suggests that the improvement in survival is unlikely to be entirely explained by increased use of adjuvant or neoadjuvant radiation therapy.

Due to limitations in the available data within the SEER program, it is difficult to accurately assess local recurrence rates after surgery or radiation therapy. Randomized controlled trials have suggested that radiation may decrease the rates of local recurrence following surgery in ESTS, particularly in high-grade tumors [8, 30]. Several studies have attempted to correlate the relative benefit of radiation therapy for local control to various patient- and tumor-related factors, including tumor grade, size, depth relative to the fascia, and surgical margin status, suggesting that each of the factors are important in determining optimal treatment [9, 31–33]. Among the limitations of the current study is the inability to fully assess margin status or to fully evaluate the local recurrence rates, as these are factors that continue to spark controversy regarding their influence on overall survival in ESTS.

The findings of this report corroborate the findings of Alamanda et al., which demonstrated marital status to be an independent predictor of overall survival in ESTS. That study included a study population that was a subset of those used for the current analysis [15]. Their findings demonstrated unmarried patients to have a lower overall survival than married patients (HR: 1.26, CI: 1.05–1.51), which are similar to our results.

A known limitation of the SEER database is the inability to account for the use of chemotherapy in the analysis. Two meta-analyses failed to find an association between adjuvant doxorubicin therapy and overall survival of soft tissue sarcoma [34, 35]. The most recent of these analyses found a statistically significant association between combined adjuvant doxorubicin combined with ifosfamide and improved overall survival. The marginal benefit demonstrated by chemotherapy in these large meta-analyses is far less than the degree of improved survival over time that was shown in the present study. As a result, the authors do not believe that chemotherapy could entirely account for the improved survival seen over the last twenty years.

Cross-sectional population-based studies such as the present study are unable to determine causality but rather elucidate associations between an explanatory variable and survival outcome. While the incidence of extremity soft tissue sarcomas has slightly increased over the study period, the current analysis demonstrates that the 5-year overall survival rates from ESTS have significantly and progressively improved over the course of twenty years, from 28% in the earliest period to 62% in the most recent. These findings are in the setting of seemingly adverse trends of increased age of the patients and more advanced stage of disease over the same time periods. The results are most striking in the older patients, with the seemingly largest improvements being seen in patients older than 30 years old.

In an extensive investigation regarding potential underlying factors that may explain these improvements, multiple independent predictors of survival were again demonstrated. However, none of these factors appear to explain the trend of improved 5-year survival in this cohort. The observation that year of diagnosis remained a significant predictor of survival on multivariate analysis suggests that the improvement in survival of ESTS from 1991 to 2010 may be due to factors that are external to this data. Further work would be beneficial to elucidate the nature of these interesting findings, in order to better understand the underlying explanations for this apparent improvement in overall prognosis, despite worsening rates of traditional prognostic factors of age, tumor size, and the presence of metastasis on presentation.

## Figures and Tables

**Table 1:** Descriptive statistics of the study population (*n* = 12,546).

Characteristic	Percent of total			*P* value^a^
	1991–1996 (*n* = 2122)	1997–2003 (*n* = 5221)	2004–2010 (*n* = 5203)	
Sex				0.1802
Male	52.6	53.7	54.9	
Female	47.4	46.3	45.1	
Site				0.0034^*^
Lower limb	71.7	73.5	75.4	
Upper limb	28.3	26.5	24.6	
Hispanic				<0.0001^*^
Non-Hispanic	90.7	88.8	87.1	
Spanish-Hispanic-Latino	9.3	11.2	13.0	
Radiation				<0.0001^*^
No	49.7	48.6	45.2	
Yes	48.8	48.8	523.0	
Unknown	1.5	2.6	1.9	
Surgery				0.31
No	8.4	7.6	8.3	
Yes	91.0	91.7	91.2	
Unknown	0.6	0.7	0.5	
Marital status				0.141
Single	16.7	18.5	17.9	
Married	58.2	57.5	58.0	
Other	21.25	19.8	20.8	
Unknown	3.9	4.2	3.4	
Age				<0.0001^*^
<30	9.2	8.0	6.8	
30–59	41.1	44.4	41.2	
60+	49.7	47.6	52.0	
Histology				<0.0001^*^
NOS	10.7	12.3	22.3	
Fibromatous	42.0	37.0	26.4	
Myxomatous	0.4	0.6	1.3	
Lipomatous	23.9	22.9	26.4	
Myomatous	11.0	13.9	13.4	
Synovial	7.5	8.3	6.4	
All other types	4.6	5.1	4.3	
Grade				<0.0001^*^
I	14.4	14.2	18.9	
II	17.2	16.6	18.2	
III	14.9	18.5	22.7	
IV	19.0	25.2	35.8	
Unknown	34.5	25.5	4.5	
Metastasis				<0.0001^*^
No	86.1	86.9	85.1	
Yes	9.1	9.4	14.9	
Unknown	4.9	3.7	0	
Tumor size				<0.0001^*^
<=5 cm	30.6	30.2	32.4	
>5 cm	46.8	49.5	64.9	
Unknown	22.6	20.3	2.7	

^*^Significant on a *P* < 0.05 level.

NOS: not otherwise specified.

^a^ *P* value for testing of significance between time periods.

**Table 2:** Univariate survival results for patients diagnosed with ESTS 1991–2010, stratified by time period.

Characteristic	Proportion 5-year survival rates					
	1991–1996 (*n* = 2122)	*P* value	1997–2003 (*n* = 5221)	*P* value	2004–2010 (*n* = 5203)	*P* value
Overall	0.28		0.40		0.62	
Sex		0.0183^*^		0.0085^*^		0.0216^*^
Male	0.25		0.38		0.60	
Female	0.30		0.42		0.65	
Site		0.0184^*^		0.0020^*^		0.0013^*^
Lower limb	0.27		0.39		0.60	
Upper limb	0.30		0.43		0.66	
Hispanic		0.4466		0.0004^*^		0.0658
Non-Hispanic	0.28		0.39		0.61	
Spanish-Hispanic-Latino	0.22		0.49		0.67	
Radiation		0.2479		0.34		0.0011^*^
No	0.31		0.41		0.61	
Yes	0.25		0.38		0.63	
Unknown	0.37		0.44		0.61	
Surgery		<0.0001^*^		<0.0001^*^		<0.0001^*^
No	0.07		0.09		0.14	
Yes	0.30		0.42		0.66	
Unknown	0.44		0.36		0.33	
Marital status		<0.0001^*^		<0.0001^*^		<0.0001^*^
Single	0.39		0.45		0.60	
Married	0.30		0.44		0.66	
Other	0.15		0.24		0.50	
Unknown	0.40		0.40		0.64	
Age		<0.0001^*^		<0.0001^*^		<0.0001^*^
<30	0.62		0.66		0.67	
30–59	0.48		0.55		0.72	
60+	0.13		0.24		0.53	
Histology		<0.0001^*^		<0.0001^*^		<0.0001^*^
NOS	0.23		0.29		0.45	
Fibromatous	0.26		0.41		0.62	
Myxomatous	NA		NA		0.64	
Lipomatous	0.34		0.53		0.80	
Myomatous	0.26		0.32		0.57	
Synovial	0.29		0.38		0.55	
All other types	0.22		0.24		0.50	
Grade		<0.0001^*^		<0.0001^*^		<0.0001^*^
I	0.40		0.67		0.89	
II	0.39		0.51		0.80	
III	0.17		0.30		0.57	
IV	0.17		0.26		0.48	
Unknown	0.28		0.40		0.14	
Metastasis		<0.0001^*^		<0.0001^*^		<0.0001^*^
No	0.30		0.44		0.70	
Yes	0.03		0.06		0.14	
Tumor size		<0.0001		<0.0001^*^		<0.0001^*^
<=5 cm	0.38		0.53		0.75	
>5 cm	0.20		0.31		0.58	
Unknown	0.31		0.43		0.11	
County		0.6359		<0.0001^*^		0.1131
Metro county	0.28		0.41		0.62	
Non-Metro county	0.27		0.30		0.59	
Geographic region		1		<0.0001^*^		0.3004
Midwest	0.29		0.44		0.62	
Northeast	0.27		0.28		0.61	
South	0.32		0.31		0.59	
West	0.26		0.44		0.63	
Race		0.0719		0.0002^*^		0.0332^*^
African American	0.29		0.36		0.54	
Caucasian	0.26		0.39		0.63	
Other	0.37		0.47		0.60	

^*^Significant on a *P* < 0.05 level.

NOS: not otherwise specified.

**Table 3:** Hazard ratios and 95% confidence intervals from multivariate analysis of a 20-year period.

Characteristic	Hazard ratio	95% CI	*P* value
Year of diagnosis			
2004–2010		Reference group	
1991–1996	3.021	2.775, 3.290	<0.0001^*^
1997–2003	2.212	2.063, 2.371	<0.0001^*^
Sex			
Male		Reference group	
Female	0.808	0.762, 0.856	<0.0001^*^
Tumor location			
Lower limb		Reference group	
Upper limb	0.940	0.880, 1.004	0.0657
Hispanic ethnicity			
Non-Hispanic		Reference Group	
Hispanic	0.852	0.770, 0.942	0.0217^*^
Radiation^a^			
No		Reference Group	
Yes	0.799	0.753, 0.848	<0.0001^*^
Surgery^a^			
No		Reference Group	
Yes	0.436	0.399, 0.476	<0.0001^*^
Marital status^a^			
Married		Reference Group	
Other	1.473	1.374, 1.578	<0.0001^*^
Single	1.451	1.322, 1.580	<0.0001^*^
Age			
<30		Reference Group	
30–59	1.698	1.462, 1.972	<0.0001^*^
60+	3.926	3.371, 4.573	<0.0001^*^
Geographic region			
West		Reference Group	
Midwest	0.937	0.850, 1.032	0.1849
Northeast	1.103	1.008, 1.208	0.0323
South	0.998	0.898, 1.109	0.9740
Histology			
Lipomatous		Reference Group	
All other types	1.380	1.201, 1.586	<0.0001^*^
Fibromatous	1.119	1.019, 1.228	0.0187^*^
Myomatous	1.266	1.134, 1.413	<0.0001^*^
Myxomatous	0.982	0.667, 1.445	0.9267
NOS	1.567	1.410, 1.740	<0.0001^*^
Synovial	1.708	1.493, 1.954	<0.0001^*^
Grade^a^			
I		Reference Group	
II	1.557	1.352, 1.793	<0.0001^*^
III	2.581	2.260, 2.947	<0.0001^*^
IV	2.883	2.533, 3.282	<0.0001^*^
Metastasis			
No		Reference Group	
Yes	3.320	3.074, 3.587	<0.0001^*^
Tumor Size^a^			
<=5 cm		Reference Group	
>5 cm	1.743	1.620, 1.876	<0.0001^*^
% Foreign born^b^	0.992	0.988, 0.995	<0.0001^*^
% < HS education^b^	1.014	1.010, 1.019	<0.0001^*^

^*^Significant on a *P* < 0.05 level.

NOS: not otherwise specified.

^a^HRs for variables with “unknown” values are not included in these results.

^b^High school graduation is based upon county-level 2000 census data.
